# Ambiguities in dietary antioxidant supplementation compared to calcium channel blockers therapy

**DOI:** 10.3389/fphar.2015.00010

**Published:** 2015-02-03

**Authors:** Théophile Godfraind, Salvatore Salomone

**Affiliations:** ^1^Faculté de Médecine et de Dentisterie, Université Catholique de LouvainBrussels, Belgium; ^2^Department of Biomedical and Biotechnological Sciences, Catania UniversityCatania, Italy

**Keywords:** antioxidants, calcium channel blockers, vitamin E, amlodipine, lacidipine, nifedipine, therapy, drug safety

Antioxidants are widely considered as being essential for an optimal health so that supplementation with natural antioxidants is advised for cancer, cardiovascular diseases and other various pathologies, whereas supplementation with vitamin pills may not be advised, as discussed below. By clicking “antioxidant miracle” on a web search engine one gets about 2 million results. This mainly relates to vitamin E and C, the former being lipid-soluble and the latter being water-soluble, which are best known by laypeople. Antioxidants neutralize the harmful effects of excessive formation of reactive oxygen species (ROS) (Godfraind, 2004). They share this quality with major therapeutic agents such as dihydropyridine-type calcium channel blockers (Godfraind, 2004) that are established drugs for treating cardiovascular diseases. In this opinion paper, we intend to compare some cardiovascular outcomes of dietary antioxidant supplements versus calcium channel blockers (CCBs).

Both clinical and experimental evidences indicate that ROS play an important role in the development of several diseases including cancer (Chandel and Tuveson, 2014) and hypertension (Godfraind, 2004). The mitochondrial electron transport chain and NADPH oxidases (NOXs) localized to the plasma membrane and the membrane of cellular organelles are producers of reactive oxygen species. NOXs enzymes are a family of heme-containing transmembrane proteins (Bedard and Krause, 2007). These enzymes transport electrons across the membrane from a cytosolic electron donor to an electron acceptor located in the extracellular or in the luminal space. NADPH serves as electron donor and oxygen as electron acceptor for the production of reactive oxygen species (Bedard and Krause, 2007). ROS comprise superoxide anion (O2^·−^), hydroxyl radical (HO^·^), peroxyl radical (RO^·^_2_), alkoxyl radical (RO^·^) (Dunford, 1987).

The highly reactive hydroxyl radical can react with molecules in cells, including DNA, proteins and lipids (Girotti, 1998). In the process of peroxidation, hydroxyl radical reaction with phospholipids is forming water (Girotti, 1998). For example, assuming that LH represents the lipid and OH^·^ the hydroxyl radical (the ^·^ representing an electron):

$$\text{LH}+{\text{OH}}^{\cdot }\to {\text{L}}^{\cdot }+{\text{H}}_{2}\text{O}.$$

The lipid radical (L^·^) can then propagate a chain of reactions causing breakdown of a number of phospholipids and so damaging cell membrane and cellular function (Girotti, 1998). Endogenous antioxidants (e.g., glutathione) and antioxidant vitamins (e.g., vitamin E) have been shown to stop these reactions (Girotti, 1998). Therefore, they may prevent peroxidative damage to membranes and other cellular molecules, including proteins and DNA (Girotti, 1998; Jialal et al., 2002).

When considering the cardiovascular system, excess production and/or impaired break-down of O2^·−^, as well as a decrease in NO bioactivity produce oxidative stress (Mangge et al., 2014). Among consequences, one observes decreased NO bioavailability in the vasculature, stroke and cardiovascular remodeling. In human hypertension, biomarkers of systemic oxidative stress are elevated (Redon et al., 2003). Among biomarkers, ROS may be directly measured by electron spin resonance, thiobarbituric acid reactive substances (TBARS) may be assessed by colorimetric or fluorometric methods, other biomarkers may be assessed by ELISA.

Agents exerting antioxidant activity comprise two major classes: enzymes and scavengers. Antioxidants enzymes consist of superoxide dismutase (SOD), catalase and glutathione peroxidase. Dismutation of O2^·−^ by SOD produces H_2_O_2_ a more stable ROS, which in turn is converted into H_2_O by catalase and glutathione peroxidase (de Haan et al., 1992). Scavengers react with ROS, preventing the oxidation reaction from continuing. Among scavengers listed in Box (Godfraind, 2004) the most used as dietary supplements and the most studied are vitamins E and C. According to several authors the antioxidant properties of CCBs may be attributed either to direct scavenging effect or to preservation of the activity of superoxide dismutase. It has been experimentally shown that some CCBs may inhibit oxidative damage to lipids associated with cellular membranes and lipoprotein particles. Under controlled experimental conditions, they may inhibit lipid peroxide formation at concentrations as low as 10.0 nM, an effect independent of calcium channel modulation. This antioxidant activity has been reported with high lipophilic CCBs (Mason et al., 1993; Godfraind, 2005) when their chemical structure facilitates proton-donating and resonance-stabilization mechanisms that quench the free radical reaction. When inserted into a location in the membrane near polyunsaturated fatty acids, the CCBs amlodipine and lacidipine donate protons to lipid peroxide molecules, thereby reducing the accumulation of peroxides. The remaining unpaired free electron associated with the CCB molecule can be stabilized in well-defined resonance structures linked with the dihydropyridine ring of the CCB molecule. The reaction that describes the antioxidant effects of a dihydropyridine (DHP) CCB is as follows, where LOO^·^ represents a lipid peroxide molecule (Godfraind, 2004):

$${\text{LOO}}^{\cdot }+\text{DHP}\to \text{LOOH}+{\text{DHP}}^{\cdot }$$

The use of dietary supplementation with antioxidants has been studied in several diseases, including cancer and cardiovascular pathologies. As far as cancer is concerned, as pointed out by Chandel and Tuveson in a recent commentary (Chandel and Tuveson, 2014), ROS both accelerate and delay cancer initiation and progression (Chandel and Tuveson, 2014). These authors explained these conflicting outcomes as related to the multiple roles that ROS play during the evolution of cancer cells and to their site of action. ROS can promote cancer by oxidizing specific intracellular chemical moieties, resulting in genetic mutations and/or activation of biochemical pathways that stimulate proliferation and neoplastic transformation (Chandel and Tuveson, 2014). On the other hand, the therapeutic effect of ionizing radiation and of many common chemotherapeutic drugs in the treatment of cancer depends on the cytotoxic action of ROS. Chandel and Tuveson (2014) proposed that this dual action could result from whether or not the site of action of ROS is distant from mitochondria. ROS specifically oxidize certain spatially collocated proteins to activate a variety of cancer-stimulating signaling pathways (Chandel and Tuveson, 2014). Distant from the mitochondria and NADPH oxidases, ROS nonspecifically damage macromolecules (DNA, RNA, lipids, and proteins) and are toxic to both normal and cancer cells (Chandel and Tuveson, 2014). Dietary antioxidants neutralize excess of reactive oxygen species at distance of the site of their production while leaving unperturbed the generated tumorigenic ROS (Chandel and Tuveson, 2014). Accordingly, their pharmacological effect might be anti- or pro-cancer development.

Human studies have shown that, in acute conditions (Donato et al., 2010), a cocktail of antioxidant vitamins and α-lipoic acid (AOC: vitamins C, E and α-lipoic acid) restored the attenuated vasodilatation due to handgrip exercise in old subjects, in agreement with reduced blood venous levels of lipid peroxide (Donato et al., 2010). But AOC had a different effect in young and trained old subjects, wherein it reduced the handgrip exercise induced vasodilatation despite the AOC reduction in free radicals (Donato et al., 2010). According to the authors of this report, their observation suggests that exercise training in older subjects alters the balance between pro- and antioxidant forces, resulting in a greater reliance on handgrip-mediated vasodilation in the exercise-trained state (Donato et al., 2010). This indicates that free radicals efficacy might be affected by local processes within the tissue, when comparing physiological responses to a same exercise in a given vascular territory.

Animal studies have shown that treatment with superoxide dismutase (SOD) mimetics or antioxidants, according to the drug dosage, improves vascular and renal function, regresses vascular remodeling, and reduces blood pressure (Godfraind, 2005). Stroke is one of the most harmful complications of hypertension (Moskowitz et al., 2010). Studies comparing two strains of hypertensive rats and measuring oxidative stress markers, including total antioxidation capacity (TAC), glutathione peroxidase (GPx) activity, and malondialdehyde (MDA), have confirmed that oxidative stress is increased in experimental cerebral ischemia (Zhang et al., 2011). This is in agreement with our previous observations in spontaneously hypertensive stroke- prone rats (SHRSP) subjected to salt load (Napoli et al., 1999), which causes animal death by acute stroke. In this study (Napoli et al., 1999) we compared the protective effect of vitamin E to the dihydropyrine-type CCBs lacidipine and nifedipine, which are endowed with antioxidant action. As shown in Figure 1, rats exposed to high salt diet died from the 7th week of exposure, so that all animals were dead after the 10th week. In rats treated with vitamin E, the death curve was displaced to the right illustrating the effect of vitamin E, which reduced mortality. In the groups treated by CCBs there was no mortality during the 16 weeks of salt loading observation. Biochemical and histochemical markers of oxidative stress were increased by salt load and similarly reduced by drug treatments (Napoli et al., 1999). It is likely that the antioxidant action of vitamin E could delay but not overcome the adverse effects on survival in this experimental setting, while other additional mechanisms discussed elsewhere (Godfraind, 2005), including effects on lipid oxidation, are involved in the protective effect of CCBs.

**Figure 1:**
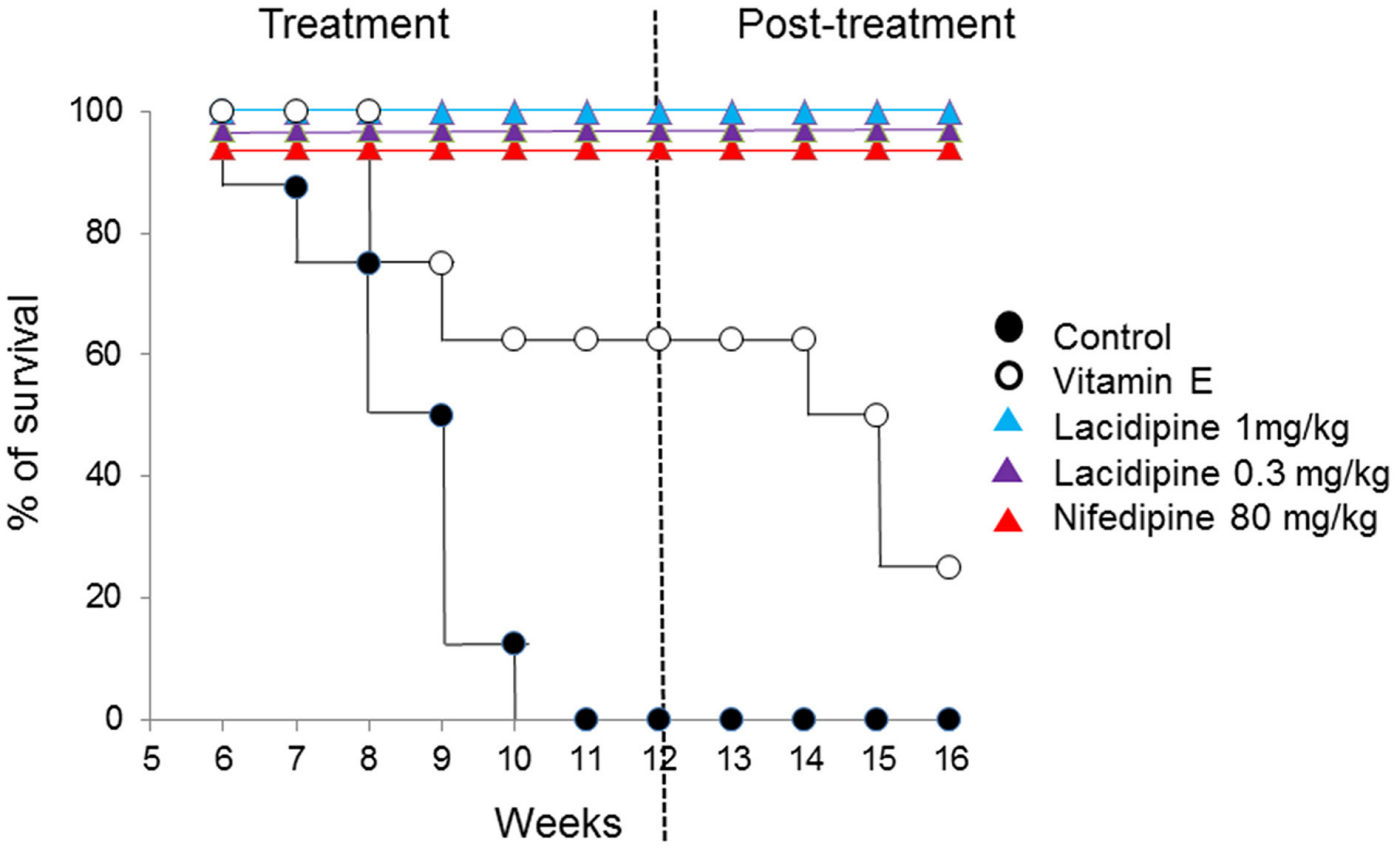
**Prolongation of survival in salt-loaded, stroke-prone spontaneously hypertensive rats (SHR-SP) with high mortality due to malignant hypertension by CCBs or vitamin E (redrawn from Napoli et al., 1999)**.

Experimental studies summarized in this opinion paper have shown how antioxidants inactivated reactive oxygen species allowing reduction of their tissue deleterious effects. *In vivo* animal experiments reported above on experimental stroke illustrated that for a similar reduction of oxidative stress indicators, the antioxidant vitamin E and CCBs were not endowed with identical mortality-reducing effects (Napoli et al., 1999). Indeed, vitamin E just delayed mortality of salt-loaded stroke-prone SHRs when CCBs treated rats did survive salt load (Napoli et al., 1999). This indicates that the protective effect of vitamin E in this animal experimental set up if not inexistent is extremely weak (Napoli et al., 1999). It could be argued that short- term animal experiments could not be predictive of therapeutic action in Humans with long-term use of antioxidants. Therefore we selected clinical studies of long duration considering a sufficiently large number of patients in order to obtain evidence-based conclusions. Wang et al. reported results of a meta-analysis (Wang et al., 2007) designed to obtain a quantitative overview of outcome trials on the efficacy of amlodipine or angiotensin receptor blockers (ARBs) in the prevention of stroke and myocardial infarction. Authors studied amlodipine versus placebo or other antihypertensive drugs including ARBs in major multicenter randomized clinical trials (Wang et al., 2007). The analysis including seven trials with 78,323 randomly assigned patients indicated that CCBs offer the best statistically significant protection against stroke and myocardial infarction (Wang et al., 2007). Furthermore it appeared that some outcomes were not directly related to reduction of blood pressure, which supports an additional beneficial role of CCBs as antioxidants in stroke and CVD, as suggested elsewhere (Godfraind, 2004). No doubt that such an action of CCBs may reduce mortality, but, to date, their antioxidant action has not been evaluated in randomized clinical trials. Nevertheless, RCTs confirmed in humans the protective effect of CCBs observed in some experimental animal studies (Napoli et al., 1999). Since there are no comparative randomized clinical studies comparing CCBs and antioxidants, we could only use meta-analyses including RCTs. Schurks et al. conducted a meta-analysis on human studies (Schurks et al., 2010). They included nine randomized controlled trials investigating the effect on stroke of dietary supplementation with vitamin E at daily doses between 50 and 200 mg. From an analysis involving 118,765 participants the authors concluded that vitamin E increased the risk for hemorrhagic stroke by 22% and reduced the risk of ischemic stroke by 10%. This differential risk pattern appeared obscured when looking at the cumulative risk of stroke (Schurks et al., 2010). Given the relatively small risk reduction of ischemic stroke and the generally more severe outcome of hemorrhagic stroke, author's conclusion was that indiscriminate widespread use of vitamin E should be cautioned against. A Cochrane meta-analysis (Bjelakovic et al., 2012) studied the beneficial and harmful effects of antioxidant supplements for prevention of mortality in adults. The authors included all primary and secondary prevention randomized clinical trials on antioxidant supplements (beta-carotene, vitamin A, vitamin C, vitamin E, and selenium) versus placebo or no intervention (Bjelakovic et al., 2012). The analysis included 78 randomized trials with 296,707 participants. The mean age was 63 years and the mean proportion of women equaled 46%. All antioxidants were administered orally, either alone or in combination with vitamins, minerals, or other interventions. The duration of supplementation varied with a mean of 3 years and a median of 2 years. There was no significant difference in effect between the primary prevention and the secondary prevention trials (Bjelakovic et al., 2012). In the 56 trials with a low risk of bias, the antioxidant supplements even increased mortality, mainly with beta-carotene and vitamin E (Bjelakovic et al., 2012). In another publication, data of this Cochrane meta-analysis were evaluated for beta-carotene, vitamin A and vitamin E, which, for a dose above 15 mg/day, significantly increased mortality (Bjelakovic et al., 2013). It is noteworthy that in AREDS trials it was observed that antioxidants preparations (see Box 1 in Supplementary Material) reduced the risk of progression to advanced AMD (age-related macular degeneration (Age-Related Eye Disease Study 2 (AREDS2) Research Group et al., 2013).

In conclusion, at pharmacologically active concentrations, Vitamin E and CCBs have a similar effect on markers of oxidative stress in experimental studies while corresponding data in humans are scarce. However, the therapeutic outcomes are quite different. In randomized control trials (RCT) CCBs improved cardiovascular risk (Godfraind, 2014) while, with exception for AREDS trials, vitamins C and E resulted ineffective (Myung et al., 2013) or, in some situations, even detrimental (Gale et al., 1995).

Thus, there is currently no evidence that allows recommending dietary supplementation with antioxidants for the primary or secondary prevention of cardiovascular disease.

## Conflict of interest statement

The authors declare that the research was conducted in the absence of any commercial or financial relationships that could be construed as a potential conflict of interest.
