# Supplementary material for: Ambiguities in dietary antioxidant supplementation compared to calcium channel blockers therapy
Source: Front Pharmacol. 2015 Feb 3;6:10. doi: 10.3389/fphar.2015.00010 (PMC4315019; doi:10.3389/fphar.2015.00010)
Supplement: Supplementary file 1 [file Table1.DOCX]

**Box 1. Antioxidant scavengers in food supplements**

Vitamin C (ascorbic acid, water-soluble) and vitamin E (α-tocopherol, lipid-soluble) may be found in supplements, dispensed as OTC for oral administration (tablets, capsules or liquid formulations), commonly containing individual dosages of 0.5-1 g for vitamin C and of 100 -1000 UI for vitamin E (approximatively corresponding to 33-333 mg).

In the Cochrane meta-analysis discussed in the text (ref. 12), vitamins were administered daily or on alternate days as oral supplements. The dose range of vitamin C was 60 to 2000 mg (mean 459 mg; median 400 mg), the dose range of vitamin E was 10 to 5000 IU (mean 539 IU; median 400 IU), approximatively corresponding to 7 to 3330 mg (mean 359 mg; median 267 mg). Other antioxidant supplements considered here were: beta-carotene 1.2 to 50.0 mg (mean 19.2 mg; median 16 mg), vitamin A 1333 to 200,000 IU (mean 17,491 IU; median 5000 IU), and selenium 20 to 200 μg (mean 94 μg; median 75 μg).

The AREDS tablet consisted of vitamins C (500 mg), E (400 IU), and beta-carotene (15 mg). Zinc was given as zinc oxide (80 mg) along with copper as cupric oxide (2 mg) daily. AREDS 2 formulation contained in addition: lutein (10 mg), zeaxanthin (2 mg), DHA (350 mg), EPA (650 mg), or both.

*Content of vitamin C and vitamin E in vegetable preparations*

**Vitamin C** (L-ascorbic acid, from http://ods.od.nih.gov/factsheets/VitaminC-HealthProfessional/)

Recommended dietary allowance in adults: 75-120 mg (but avoid continuous automedication for > 1 year)

**Content in food:**

**Food mg vitamin C**

Red pepper, sweet, raw, ½ cup 95

Orange juice, ¾ cup 93

Orange, 1 medium 70

Grapefruit juice, ¾ cup 70

Kiwifruit, 1 medium 64

Green pepper, sweet, raw, ½ cup 60

Broccoli, cooked, ½ cup 51

Strawberries, fresh, sliced, ½ cup 49

Brussels sprouts, cooked, ½ cup 48

Grapefruit, ½ medium 39

Broccoli, raw, ½ cup 39

Tomato juice, ¾ cup 33

Cantaloupe, ½ cup 29

Cabbage, cooked, ½ cup 28

Cauliflower, raw, ½ cup 26

Potato, baked, 1 medium 17

Tomato, raw, 1 medium 17

Spinach, cooked, ½ cup 9

Green peas, frozen, cooked, ½ cup 8

**Vitamin E** (α-tocopherol, from http://ods.od.nih.gov/factsheets/VitaminE-HealthProfessional/)

Recommended dietary allowance in adults: 15 mg (but avoid continuous automedication for > 1 year)

**Content in food:**

**Food mg vitamin E**

Wheat germ oil, 1 tablespoon 20.3

Sunflower seeds, dry roasted, 1 ounce 7.4

Almonds, dry roasted, 1 ounce 6.8

Sunflower oil, 1 tablespoon 5.6

Safflower oil, 1 tablespoon 4.6

Hazelnuts, dry roasted, 1 ounce 4.3

Peanut butter, 2 tablespoons 2.9

Peanuts, dry roasted, 1 ounce 2.2

Corn oil, 1 tablespoon 1.9

Spinach, boiled, ½ cup 1.9

Broccoli, chopped, boiled, ½ cup 1.2

Soybean oil, 1 tablespoon 1.1

Kiwifruit, 1 medium 1.1

Mango, sliced, ½ cup 0.7

Tomato, raw, 1 medium 0.7

Spinach, raw, 1 cup 0.6
